# Data for evaluating mine roof stability via acoustic impact

**DOI:** 10.1016/j.dib.2022.107854

**Published:** 2022-01-20

**Authors:** Travis Wiens, Shahriar Islam

**Affiliations:** Department of Mechanical Engineering, University of Saskatchewan, Saskatoon, Sk, Canada

**Keywords:** mining, safety, acoustics, impact, geology, rock mechanics, potash

## Abstract

The stability of underground mine roofs is critical to the safety of miners. They often assess the condition of the roof by striking it with a long bar and then interpret the sound of the impact as either “tight” (safe) or “drummy” (unsafe). This paper presents a dataset of a 3309 acoustic recordings of such impacts, labelled as by an expert miner as either drummy or tight. Data was recorded from a number of sites within 5 different underground mines, all potash mines in the Canadian province of Saskatchewan.

## Specifications Table

**Table:** 

Subject	Acoustics and Ultrasonics
Specific subject area	Mine safety, roof stability assessment
Type of data	Acoustic recordings
How data were acquired	Acoustic data, recorded using a Zoom H4nPro digital audio recorder and Sennheiser E 845-S microphone.
Data format	Raw
Parameters for data collection	Recording sample rate: 48 KHz Recording length: 36 000 samples
Description of data collection	For each recording, the microphone was mounted on a stand near the mine roof. The roof was then impacted by a scaling bar (a steel or aluminum bar 2-3 m in length) near the microphone, with the recorder running. Recordings were taken from various locations within each mine. Each recording was also labelled as drummy (unsafe) or tight (safe) by an expert.
Data source location	Data was recorded from 5 mine sites (identity confidential). Each are underground potash mines within the province of Saskatchewan, Canada and are situated within the Patience Lake geological member.
Data accessibility	With the article
Related research article	Travis Wiens, Shahriar Islam, Using Acoustic Impacts and Machine Learning for Safety Classification of Mine Roofs, Int. J. Rock Mechanics and Mining. In Press

## Value of the Data

• These data are important because they provide a previously unrecorded sample of a process that is critical for the safety of underground miners.
• The primary beneficiaries are employees of underground mines, who can expect safer working conditions
• This data may be used to train machine learning algorithms to automatically identify the safety of a mine roof (e.g. see [1])
• This data may be used to quantify the safety of a mine roof, rather than relying on the qualitative and subjective opinion of miners
• This will allow for safer mine operation with more confident assessment of the roof by more junior employees

## 1 Data Description

mine_impact_data_2019.mat: a Matlab format data file including the following variables:

• x: time series of recordings, with each column a separate recording
• y: expert identification for each recording (0="drummy", 1="tight")
• mine: index for one of 5 mine locations, for each recording

mine_impact_data_2019.csv: a CSV text file including the same data as above, with each column a separate recording. The first row is the mine location index, followed by the expert identification, y, followed by the recordings, x.

## 2 Experimental Design, Materials and Methods

We made 6 visits to a total of 5 potash mine sites in Saskatchewan. Each mine was situated in the Patience Lake geological member and is characterized by a vein of potash with an overlying layer of salt. The mine tunnels are bored just below this salt beam, which supports the roof of the tunnel. Generally, the salt beam is well consolidated and thick, but occasionally a clay layer will descend into the salt, weakening it. When struck by a scaling bar (a 2-3 m long steel or aluminum bar), a strong and stiff salt beam will not reverberate, producing a sound termed “tight”. If the salt beam is weak or separated from overlying rock, the sound will be hollow and is termed “drummy”. Miners listen to this acoustic information to evaluate the safety of an area before entering. However, this is a subjective evaluation and requires an experienced worker to interpret the results.

We spent a working days recording data at each underground mine site, recording between 351 and 882 impacts at each site, for a total of 3309. Locations were selected to have a variety of roof conditions, ultimately with 2212 recordings labelled “drummy” and 1097 labelled “tight”. As it is not safe to enter unsupported areas with a drummy roof, these recordings were obtained in areas where the roof was supported by roof bolts. It is believed that the roof bolts do not have a significant impact on the sound produced.

At each location, we mounted a Sennheiser E 845-S microphone on a tall microphone stand, so that the microphone was near the impact location on the roof (typically 0.3–1.0 m from the impact location to the microphone). This was required as previous trials had indicated that recording at ear height resulted in signals that were heavily influenced by the room acoustics. We activated a Zoom H4nPro digital audio recorder to record the microphone signal, recording in raw WAV format at a 48 KHz sample rate. We then asked a mine worker to strike the roof with a scaling bar in the same way that they would normally strike it when evaluating the roof. The worker then indicated whether they judged the roof to be drummy or tight, which was recorded for each impact.

After the data was recorded we processed the impact recordings into a consistent format. First, we aligned the impacts in time. For each recording, we calculated the noise floor of the recording (the standard deviation). We then identified the first sample in the recording that exceeded ten times this noise floor, indicating the start of the impact signal. Starting from a point 100 samples (2.1 ms) prior to this trigger point, we then cropped the signal to a length of 0.75 s (36 000 samples). This length ensured that all reverberations within the roof material had died out. Reverberations caused by the room acoustics may still exist after 0.75 s, but these reverberations are not believed to contain any useful information that is not contained in the direct impact signal. The resulting signals were not further filtered or processed.

## Ethics Statement

N/A

## CRediT Author Statement

**Travis Wiens:** Conceptualization, software, resources, writing, funding acquisition; **Shahriar Islam:** software, investigation, data curation.

## Declaration of Competing Interest

The authors declare that they have no known competing financial interests or personal relationships which have, or could be perceived to have, influenced the work reported in this article.

## Appendix Supplementary materials

Image, application 1

Image, application 2

## References

[1] Wiens T., Islam S. (2021). Using acoustic impacts and machine learning for safety classification of mine roofs. Int. J. Rock Mech. Min..

